# Comprehensive Analysis of Oral Squamous Cell Carcinomas: Clinical, Epidemiological, and Histopathological Insights With a Focus on Prognostic Factors and Survival Time

**DOI:** 10.7759/cureus.54394

**Published:** 2024-02-18

**Authors:** Jia Fatima, Ehda Fatima, Fatima Mehmood, Iman Ishtiaq, Muhammad Athar Khan, Hafiz Muhammad S Khurshid, Muhammad Kashif

**Affiliations:** 1 Oral and Maxillofacial Surgery, Bakhtawar Amin Medical and Dental College, Multan, PAK; 2 Oral Pathology, Bakhtawar Amin Medical and Dental College, Multan, PAK

**Keywords:** survival time, histological grading, opscc, oscc, hnscc

## Abstract

Background and objectives: Oral squamous cell carcinoma (OSCC) is one of the most common malignancies in the head and neck region. Particularly, high incidence rates are observed in South and Southeast Asia, attributed to the widespread use of the carcinogenic areca nut. This study aimed to investigate the clinical, epidemiological, and histopathological features of OSCC, identify prognostic factors impacting disease-free survival, and determine a post-diagnosis disease-free survival time of OSCC patients.

Methodology: Employing a descriptive cross-sectional design, the study conducted a thorough examination of the clinical, epidemiological, and histopathological aspects of OSCC among patients seeking care at a tertiary healthcare facility. Participants were personally interviewed if available, while information for unreachable or deceased individuals was extracted from archival patient records in the Oral and Maxillofacial Surgery Department, Bakhtawar Amin Medical and Dental College, Multan, Pakistan. Data analysis was performed with a significance level set at p ≤ 0.05.

Results: The mean age of the patients was 54.16 ± 11.1, with a notable concentration in the 41 years and above age group, indicating a significant prevalence of OSCC in this population. The data revealed a gender bias toward males, and a substantial proportion of patients, particularly those aged 41 years and above, had unfortunately passed away. Statistical analysis using the Fisher exact test showed a significant association between age groups and patients' current living status (p-value < 0.05).

Conclusion: Histopathologically, moderately differentiated OSCC was the most frequently encountered grade, and surgery emerged as the predominant treatment modality. The majority of patients studied had a survival period of three years or less, emphasizing the need for further exploration of factors influencing prognosis and treatment outcomes in OSCC.

## Introduction

Oral squamous cell carcinoma (OSCC) is a type of head and neck squamous cell carcinoma (HNSCC), which is among the most prevalent malignancies in the head and neck region, primarily linked to exposure to carcinogens from tobacco and excessive alcohol consumption [1]. Squamous cell carcinoma (SCC) accounts for approximately 90% of head and neck cancers (HNCs), originating from the epithelial lining of the oral cavity, pharynx, and larynx. According to the latest GLOBOCAN estimates in 2020, OSCC is the seventh most prevalent cancer worldwide, constituting about 4.5% of all cancer diagnoses globally. Moreover, it leads to around 450,000 deaths annually, representing approximately 4.6% of global cancer-related deaths. Globally, OSCC is more prevalent in men than in women, and it is more common in adults over 50 years of age [2]. The highest incidence rates are observed in South and Southeast Asia, particularly due to the widespread consumption of the carcinogenic areca nut [3]. The global incidence of OSCC has been on the rise in many countries, particularly among younger populations, with an anticipated 30% annual increase in incidence by 2030. This upward trend is partly attributed to lifestyle changes, such as increased alcohol consumption and tobacco use in developing nations. Additionally, the growing prevalence of human papillomavirus (HPV)-related oropharyngeal cancer contributes to this evolving pattern [2].

The characteristic symptoms of HNSCC vary depending on the anatomical location of the primary tumor and the underlying cause. In the oral cavity, cancers typically exhibit symptoms such as persistent mouth sores or ulcers. Early detection in this region often occurs when patients identify mass lesions themselves, experiencing symptoms that affect fundamental functions like eating and speaking, such as pain during chewing or difficulty in speaking (dysarthria). Primary tumors in the oropharynx tend to show symptoms later due to their concealed anatomical position. When present, indications like difficulty in eating (dysphagia), pain during swallowing (odynophagia), or ear pain (otalgia) often suggest a more advanced tumor. Laryngeal cancers commonly present with changes in voice or pronounced hoarseness, facilitating early-stage diagnosis [4]. Nasopharyngeal carcinoma is typically characterized by symptoms such as a cervical neck mass, nosebleeds (epistaxis), and unilateral nasal obstruction [5].

In HNSCC, the prognostic reliability is based on clinical staging (TNM). The pre-therapeutic anatomic extent (cTNM) is determined through clinical and radiologic examinations, such as MRI and CT scans, guiding the selection of primary treatment [6]. Primary curative modalities for locally or locoregionally confined HNSCC include resection, radiation, and systemic therapy [7,8]. The choice of treatment depends on the primary tumor's location, disease stage, and anticipated oncological and functional outcomes. For tumors with advanced tumor or nodal stages, postoperative radiation or combined chemoradiation (CCRT) reduces recurrence risk and improves survival [9,10].

Considering the intricate functions of the head and neck region, the inherent impacts of malignancy along with the expanding array of treatment options, exert a substantial influence on the health-related quality of life (HRQOL) for individuals with HNSCC. The diverse treatments and their combinations result in specific consequences, affecting physical, emotional, functional, and social aspects, as well as causing occupational dysfunction [11]. On average, the overall HRQOL experiences an 11% decline compared to pre-treatment levels and a 15% decline compared to years 1 and 2 after treatment [12,13]. The study's primary goals were to determine clinical, epidemiological, and histopathological features of OSCC affecting the survival time of OSCC patients treated at Bakhtawar Amin Hospital from 2017 to 2023. These findings are expected to contribute valuable information to local epidemiological and clinical data, enhancing the existing database.

## Materials and methods

Research protocol

This research was conducted at the Department of Oral and Maxillofacial Surgery in Bakhtawar Amin Medical and Dental College, Multan, Pakistan, from July 2023 to January 2024. Employing a descriptive cross-sectional design, the study aimed to perform a comprehensive audit of the clinical, epidemiological, and histopathological aspects of OSCC among patients seeking care at a tertiary healthcare facility. To uphold ethical standards, the research obtained approval from the Institutional Research/Review Board (IRB) of Bakhtawar Amin Dental College and Hospital (No. 66/2023/COD). The study meticulously adhered to the guidelines outlined in the WMA Declaration of Helsinki to ensure the confidentiality and ethical treatment of patients. Deceased patients' records were accessed from the archival patient records of the Oral and Maxillofacial Surgery Department.

Data collection

A specifically designed proforma containing specific variables was developed to select participants for the study. Individuals who were available were personally interviewed, while those unreachable or deceased had their information gathered from the archival patient records of the Oral and Maxillofacial Surgery Department. The study subjects were determined based on defined inclusion and exclusion criteria. Inclusion criteria encompassed patients of any gender, individuals aged 20 years and above, and those diagnosed with primary OSCC. Exclusion criteria comprised patients with a history of recurrence or previous treatment, individuals with underlying conditions such as tuberculosis or diabetes mellitus, and individuals with metastatic diseases. A total of 62 eligible patients meeting these criteria were identified, and the study's objectives were communicated to them. All information collected during the study was meticulously documented using the specially designed questionnaire to ensure precision and uniformity.

Statistical analysis

The collected data were subjected to analysis using IBM SPSS Statistics for Windows, Version 27 (Released 2020; IBM Corp., Armonk, New York). The association between categorical and descriptive variables was evaluated through chi-square and Fisher's exact tests, aiming to establish statistical connections. A significance level of *p* ≤ 0.05 was considered statistically significant.

## Results

**Figure 1:**
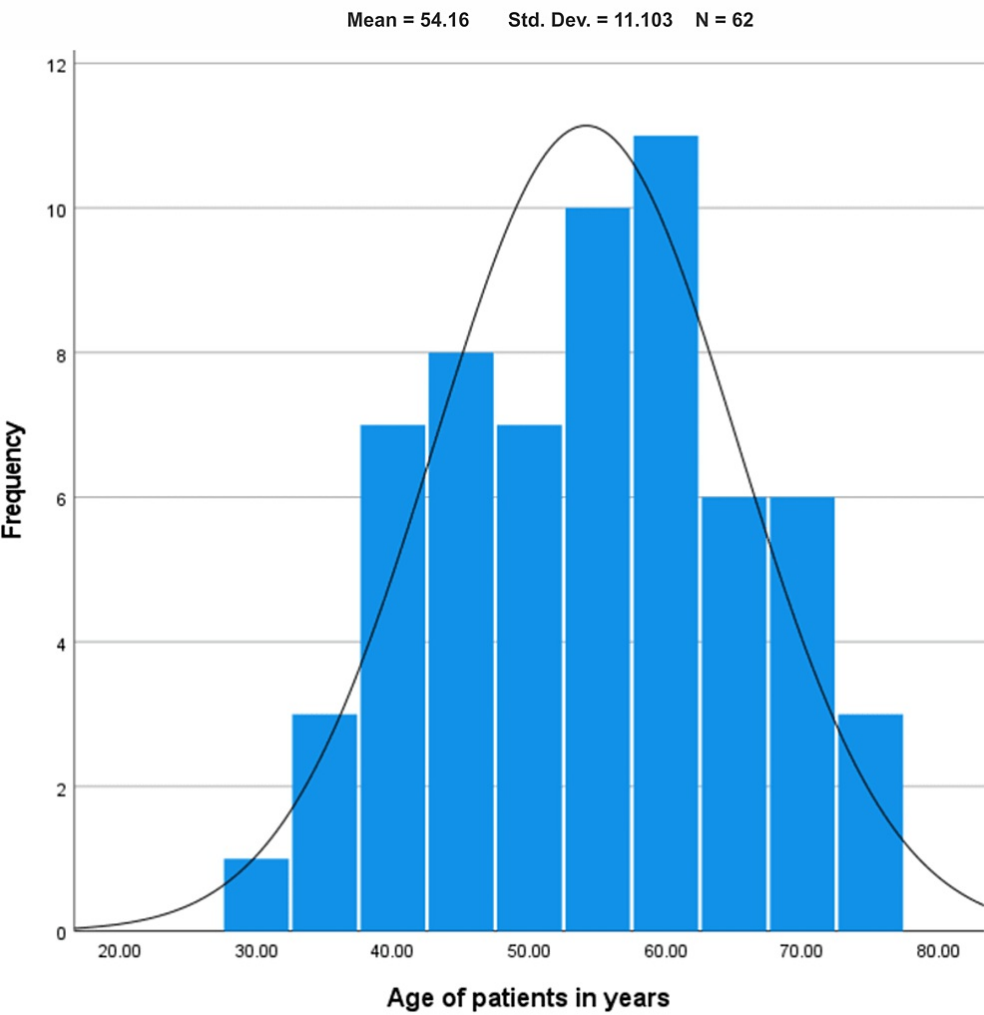
Histogram showing age-wise distribution of the study population

Figure 1 shows a histogram describing the age distribution of patients with OSCC. It illustrates that among the 62 patients included in the study, the average age of the patients was 54.16 ± 11.1. In this study, most of the subjects fell within the age range of 40 to 70 years. The histogram further depicts that the minimum age of the patients was 30 years, and the maximum age was 76 years.

Table 1 functions as a frequency distribution, providing a comprehensive overview of key demographic and clinical characteristics among the 62 patients enrolled in the study. The data delineate that, regarding the duration of diagnosis, the table reports a mean of 23.28 ± 22.7 months for 53 out of the 62 patients, signifying the average time elapsed since diagnosis. Unfortunately, data for the remaining 9 patients were not available. This information sheds light on the temporal aspect of the disease progression within the majority of the study population. Additionally, the table highlights the average tumor size for 32 patients, quantifying it at 1.90 ± 0.92 cm. Regrettably, the tumor size for the remaining 30 patients could not be documented. These insights contribute to our understanding of the age distribution, diagnosis timelines, and tumor sizes within the studied OSCC patient cohort, though limitations are acknowledged in terms of missing data for a subset of patients. 

**Table 1:** Frequency of age, duration of diagnosis, and tumor size of the patients presented to the outpatient department

	Age of patients (years)	Duration of diagnosis (months)	Tumor size (cm)
Valid number of cases	62	53	32
Missing cases	0	9	30
Mean	54.1613	23.2830	1.9063
Median	55.0000	17.0000	2.0000
Standard deviation	11.10338	22.73119	0.92838
Skewness	-0.033	2.450	0.195
Standard error of skewness	0.304	0.327	0.414
Range	46.00	131.00	2.00
Minimum	30.00	1.00	1.00
Maximum	76.00	132.00	3.00

**Figure 2:**
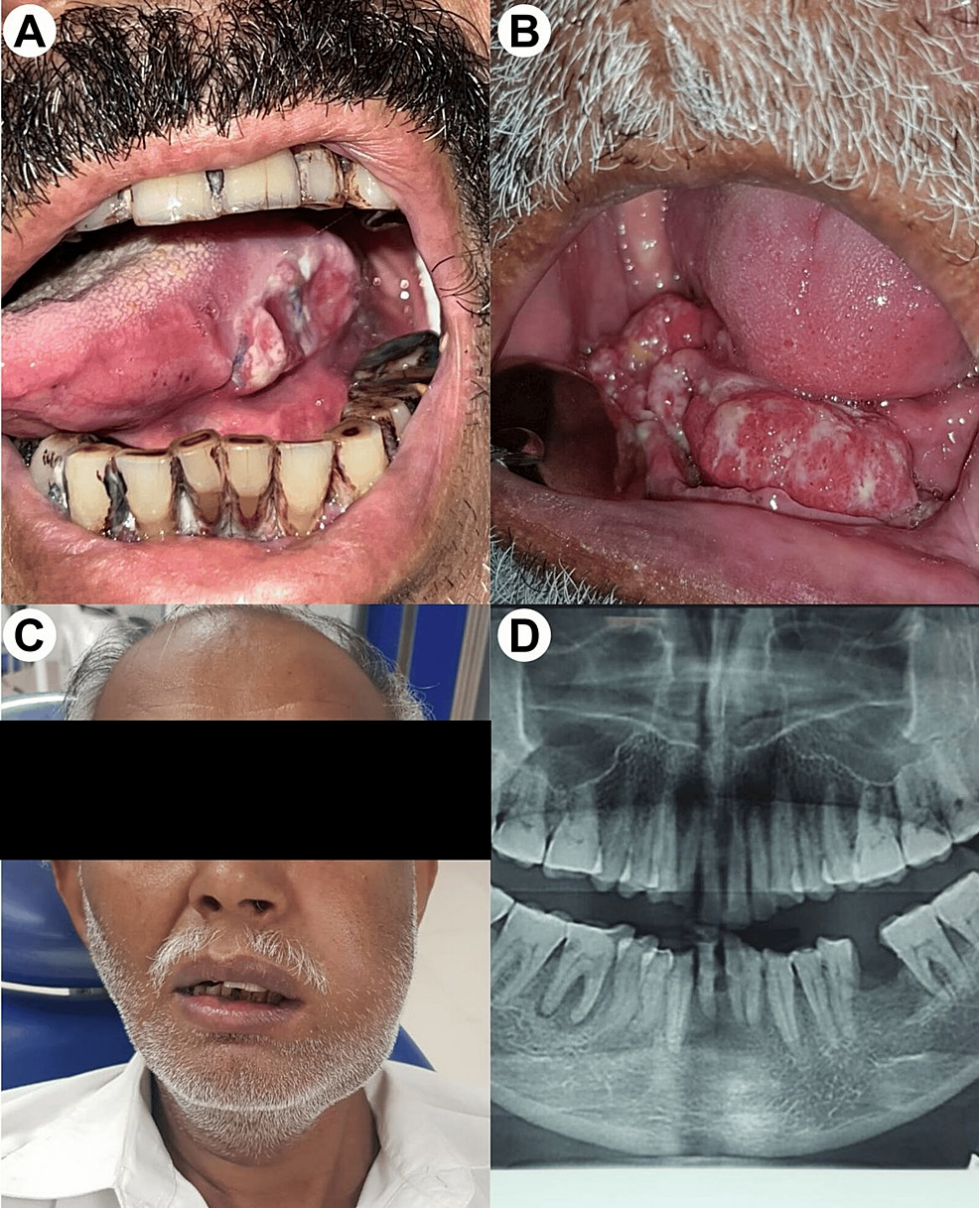
Clinical and radiographic presentation of OSCC. (A) A chronic paan (betel quid) chewer with an ulcerated mass involving the posterior-lateral border, base of the tongue, and floor of the mouth. (B) A large exophytic mass involving the mandibular edentulous ridge with surface ulceration. (C) A hard buccal mucosal and alveolar ridge mass, clinically visible as extra-oral swelling. (D) An OPG (orthopantomogram) of the same patient showing radiolucency and erosion of alveolar bone at the extracted socket area.

Table 2 provides a detailed breakdown of key characteristics among the patients in the study. Notably, the majority of patients were ≤40 years old, with males constituting 69.4% of the cohort. The predominant site of the lesion was the tongue, accounting for 41.9% of cases (Figure 2). In terms of biopsy procedures, incisional biopsy was performed on 37.1% of patients, while 41.9% underwent excisional biopsy. The mortality statistics reveal that 59.7% of the 62 patients are deceased, while 30.6% are still alive. Histologically, the most prevalent finding was moderately differentiated SCC (43.5%), followed by poorly differentiated SCC in 17.7% of patients. Well-differentiated SCC was identified in 9.7% of cases. The table concludes by highlighting that the predominant mode of treatment was surgery, administered to 83.9% of patients.

**Table 2:** Frequency of age groups, patient gender, mortality status, site of lesion, type of biopsy, histological grade, and type of treatment of the patients under study

Variables	Frequency (percentage)
Age groups	
≤40 years	9 (14.5%)
41 years and above	53 (85.5%)
Patient gender	
Male	43 (69.4%)
Female	19 (30.6%)
Mortality status	
Alive	19 (30.6%)
Deceased	37 (59.7%)
Site of lesion	
Buccal mucosa	16 (25.8%)
Tongue	26 (41.9%)
Retromolar area	3 (4.8%)
Floor of mouth	3 (4.8)
Palate	2 (3.2%)
Lips	5 (8.1%)
Type of biopsy	
Incisional	23 (37.1%)
Excisional	26 (41.9%)
Histological grade	
Well-differentiated	6 (9.7%)
Moderately differentiated	27 (43.5%)
Poorly differentiated	11 (17.7%)
Type of treatment	
None	1 (1.6%)
Surgery	52 (83.9%)
Surgery + chemotherapy	5 (8.1%)
Surgery + chemotherapy + radiotherapy	1 (1.6%)

Following a cross-tabulation analysis (Table 3), a noteworthy observation emerged: a substantial proportion of patients, particularly those aged 41 years and above, had unfortunately passed away. Subsequent application of the Fisher exact test to explore the statistical relationship between age groups and patients' current living status revealed a significant association, denoted by a p-value of <0.05. In the cross-tabulation of age groups with the site of the lesion, a prevalent trend emerged wherein most OSCC lesions were intraoral and more common among patients aged 41 years and above. Despite this, the Fisher exact test indicated a non-significant association between age groups and the site of the lesion, with a p-value exceeding 0.05. A higher proportion of moderately differentiated OSCC was observed in patients aged 41 and above, although the difference was not statistically significant (p > 0.05). Additionally, an examination of age groups and the duration of diagnosis revealed that a majority of OSCC patients, particularly those aged 41 years and above, had a diagnosis duration of three years or less, with no statistically significant difference (p > 0.05).

**Table 3:** Comparison of age groups with respect to the site of lesion, histological grade, duration of diagnosis, and current living status *p-value <0.05 is considered statistically significant.

Variables	Age groups		p-value
	≤40 years	≥41 years	
Lesional site			0.334
Intra-oral	7 (13.7%)	44 (86.3%)	
Extra-oral	0 (0.0%)	5 (100%)	
Histological grade			0.437
Well-differentiated	1 (2.3%)	5 (11.4%)	
Moderately differentiated	5 (11.4%)	22 (50.0%)	
Poorly differentiated	0 (0.0%)	11 (25.0%)	
Duration of diagnosis			0.565
≤3 years	7 (17.1%)	34 (82.9%)	
>3 years	2 (16.7%)	10 (83.3%)	
Current living status			0.014*
Alive	6 (31.6%)	13 (68.4%)	
Deceased	2 (5.4%)	35 (94.6%)	

## Discussion

A thorough review of worldwide trends in the occurrence of HNCs shows differences in the frequency of these cancers among various countries. Our study, concentrating on age-related trends, suggests that the typical age of individuals diagnosed with OSCC is 54.1 ± 11.1 years, primarily falling within the age range of 40 to 70 years. These findings align with research conducted in India, where the mean age was reported as 54.4 ± 10.2 years [14]. Johnson et al. observed that the average age of onset for OSCC in individuals of Asian descent occurred between the fifth and sixth decades of life [15]. A study in eastern India reported a mean age at OSCC diagnosis of 52.1 years [16]. According to data from the SEER program of the US National Cancer Institute, oral cancer is typically diagnosed at an average age of 65 years [17]. In our study, the most affected age group was 40-70 years, with the youngest patient being 30 years old and the oldest 76 years old. Notably, Mathew et al. suggest that in developing nations, oral cancer may exhibit a higher incidence among younger individuals compared to Western countries, possibly linked to widespread tobacco chewing, especially among young adults [18]. The consistent rise in oral cancer incidence among young men and women is likely associated with the prevalent addiction to tobacco, which is readily available at affordable prices in grocery stores and paan, or betel quid, kiosks in our country. It is crucial to highlight that the accessibility and affordability of tobacco products contribute significantly to the widespread adoption of this harmful habit.

Our research unveils a significant gender gap in the incidence of OSCC, with a higher frequency observed in males (69.4%) compared to females (30.6%). This finding aligns with existing literature, which documents male-to-female ratios ranging from 2:1 to 4:1 [19]. In India, the reported rate of oral cancer is 20 per 100,000 men. The increased prevalence among males in this context is attributed to the widespread availability and accessibility of tobacco and alcohol products, coupled with a greater propensity among men to partake in behaviors such as smoking, tobacco chewing, and alcohol consumption [20]. This higher incidence in males may be linked to their greater likelihood of engaging in behavioral risk factors, including alcohol consumption and smoking. Notably, even after adjusting for these factors, men continue to exhibit a higher incidence of cancer [21].

Our study identifies the intraoral region, specifically the tongue, as the most prevalent site for OSCC, constituting 41.9% of the lesions, particularly in patients aged 41 years and above. The second most common site, as indicated by our findings, was the buccal mucosa. These results align with existing literature, which establishes the tongue not only as the primary site among various subsites of oral cavity cancer but also as a predominant location for OSCC cases in northern and western India [22]. Additionally, our study concurs with reports that identify the buccal mucosa as a common sub-site of OSCC in the Indian population [23]. Similar trends are observed in the United States, where the oral tongue is the most frequently affected intraoral site for HNSCC, contributing to 7,100 new cases annually and representing 25-40% of all OSCC cases [24]. Notably, heavy smoking and alcohol use emerge as the two significant independent risk factors for the development of tongue SCC [25]. These findings underscore the importance of understanding regional variations and risk factors in the context of OSCC incidence, facilitating targeted preventive and therapeutic interventions.

The outcomes of our investigation unveiled that the predominant histological grade of OSCC was moderately differentiated, accounting for 43.5% of cases. The second most prevalent histological grade observed was poorly differentiated OSCC, constituting 17.7% of the cases. A local study conducted in Pakistan also identified moderately differentiated HNSCC as the most common histological grading, with poorly differentiated findings being the second most prevalent [26]. Our results align with another study in Bangladesh, which similarly reported that the majority of patients were classified as having moderately differentiated OSCC [27]. The convergence of these findings across diverse regions underscores the prevalence of moderately differentiated HNSCC as a common histological grade, with variations in the ranking of other grades reflecting potential regional differences in tumor characteristics.

Our study reveals that a significant proportion of patients experienced a survival time of ≤3 years, with the majority of individuals in this category being aged 41 years and above. This observation aligns with findings from a South American study, which reported a three-year overall survival rate of <50% for hypopharyngeal and oropharyngeal cancers, and slightly more than 50% for laryngeal and oral cavity cancers. Notably, late-stage diagnosis emerged as a robust independent predictor of poorer survival across all HNSCC sites [28]. Furthermore, our study underscores the independent association between older age at diagnosis and lower survival rates across all cancer sites. This finding is consistent with previous research conducted in Europe and North America [29,30]. The convergence of our study results with these broader trends emphasizes the universal impact of factors such as late-stage diagnosis and older age on the prognosis of OSCC. These insights underscore the importance of early detection strategies and tailored interventions, particularly for older individuals, to improve overall survival rates in OSCC cases.

While this study contributes crucial local data on epidemiological, clinical, and prognostic factors in OSCC patients, it is important to acknowledge certain limitations. These include a relatively small sample size, a combination of retrospective and prospective designs of research, incomplete data for certain variables, and reliance on information provided by patients or their attendants. To enhance the robustness of future investigations, it is recommended to involve a larger cohort of subjects and incorporate prospective data collection methods.

## Conclusions

In conclusion, our research sheds light on key demographic and clinical aspects of OSCC. The age-related findings of the current study indicate a noteworthy prevalence of OSCC in the middle-aged population in Pakistan. The data underscore a gender predilection toward males. Histopathologically, moderately differentiated OSCC was the most frequently encountered grade. Notably, surgery emerged as the predominant treatment modality. The majority of the studied patients had a survival period of three years or less, highlighting the need for further exploration of factors influencing prognosis and treatment outcomes in OSCC.
